# Accumbal Cholinergic Interneurons Differentially Influence Motivation Related to Satiety Signaling

**DOI:** 10.1523/ENEURO.0328-16.2017

**Published:** 2017-05-09

**Authors:** Teemu Aitta-aho, Benjamin U. Phillips, Elpiniki Pappa, Y. Audrey Hay, Fiona Harnischfeger, Christopher J. Heath, Lisa M. Saksida, Tim J. Bussey, John Apergis-Schoute

**Affiliations:** 1 Department of Pharmacology, University of Cambridge, Cambridge CB2 1PD, UK; 2 Department of Pharmacology Faculty of Medicine, University of Helsinki, Helsinki 00014, Finland; 3 Department of Psychology and Behavioural and Clinical Neuroscience Institute, University of Cambridge, Cambridge, CB2 3EB, UK; 4 Department of Physiology, Development, and Neuroscience, University of Cambridge, Cambridge CB2 3DY, UK; 5 School of Life Health and Chemical Sciences, the Open University, Walton Hall, Milton Keynes MK7 6AA, UK; 6 Molecular Medicine Research Laboratories, Robarts Research Institute and Department of Physiology and Pharmacology, Schulich School of Medicine and Dentistry, Western University, London, ON, Canada; 7 Brain and Mind Institute, Western University, London, ON, Canada; 8 Department of Neuroscience, Psychology, and Behaviour, University of Leicester, Leicester LE1 7RH, UK

## Abstract

Satiety, rather than all or none, can instead be viewed as a cumulative decrease in the drive to eat that develops over the course of a meal. The nucleus accumbens (NAc) is known to play a critical role in this type of value reappraisal, but the underlying circuits that influence such processes are unclear. Although NAc cholinergic interneurons (CINs) comprise only a small proportion of NAc neurons, their local impact on reward-based processes provides a candidate cell population for investigating the neural underpinnings of satiety. The present research therefore aimed to determine the role of NAc-CINs in motivation for food reinforcers in relation to satiety signaling. Through bidirectional control of CIN activity in mice, we show that when motivated by food restriction, increasing CIN activity led to a reduction in palatable food consumption while reducing CIN excitability enhanced food intake. These activity-dependent changes developed only late in the session and were unlikely to be driven by the innate reinforcer strength, suggesting that CIN modulation was instead impacting the cumulative change in motivation underlying satiety signaling. We propose that on a circuit level, an overall increase in inhibitory tone onto NAc output neurons played a role in the behavioral results, as activating NAc-CINs led to an inhibition of medium spiny neurons that was dependent on nicotinic receptor activation. Our results reveal an important role for NAc-CINs in controlling motivation for food intake and additionally provide a circuit-level framework for investigating the endogenous cholinergic circuits that signal satiety.

## Significance Statement

The decrease in the drive to eat is not all or none but instead develops over the course of a meal, where with each bite the incentive value of food is reduced, eventually resulting in the state of being sated. Such reappraisal of value is a process that has been strongly attributed to nucleus accumbens function and provides a motivation-based framework for investigating the neural underpinnings of satiety. Nicotine, by acting on central nicotinic acetylcholine receptors, is well know to be a potent anorectic; nevertheless precisely how cholinergic brain circuits regulate appetite is poorly understood. The aim of this study was therefore to determine the endogenous cholinergic circuits that underlie satiety signaling.

## Introduction

The need for maintaining energy homeostasis has resulted in the intricate wiring between specialized brain circuits for guiding food-seeking behavior. The nucleus accumbens (NAc), located in the ventral region of the striatum, is strategically located for integrating information relevant to such behavior, as it receives significant inputs from energy-sensing brain regions and ones that encode for the value of environmental stimuli (Phillipson and Griffiths, 1985; McDonald, 1991; Brog et al., 1993; Trivedi et al., 1998). By virtue of its output to motor systems, the NAc is at an interface between these regions and those necessary for generating complex actions important for survival (Jones and Mogenson, 1980; Mogenson et al., 1980).

Although comprising < 3% of striatal neurons (Bolam et al., 1984; Phelps et al., 1985; Contant et al., 1996; Zhou et al., 2002), cholinergic interneurons (CINs) are known to form a dense plexus of local innervation (Bolam et al., 1984; Kawaguchi, 1993; Contant et al., 1996; Descarries and Mechawar, 2000). This small but far-reaching NAc population is known to be critical for NAc function (Hoebel et al., 2007; Williams and Adinoff, 2008; Mark et al., 2011). For example, disruptions in NAc-CIN functioning have been linked to a number of psychiatric conditions including depression (Chau et al., 2001; Warner-Schmidt et al., 2011), addiction (Hikida et al., 2001; Itzhak and Martin, 2002; Williams and Adinoff, 2008), and related withdrawal symptomology (Hikida et al., 2003; Avena et al., 2008), highlighting the importance of this NAc neuronal population in various reward-based cognitive processes.

One theory of CIN function posits that by reducing the incentive value of rewarding stimuli, locally released NAc acetylcholine (ACh) can act as a motivational stop signal (Hoebel et al., 2007). Support for this idea comes from data demonstrating a behavioral link between increased NAc cholinergic transmission and reduced seeking behavior for drugs (Hikida et al., 2001, 2003; Zhou et al., 2007) and natural rewards (Rada et al., 2005; Avena et al., 2006). The decreased desire for food associated with satiety is not typically an all-or-none process but instead develops cumulatively over the course of a meal. The gradual change in consumption behavior occurs through an iterative devaluation of the incentive value of food. Such reappraisal of value is a process that has been strongly attributed to NAc function (Corbit et al., 2001; Cardinal et al., 2002; Singh et al., 2010; Mannella et al., 2013) and provides a motivation-based framework for investigating the neural underpinnings of satiety.

Support for NAc-ACh as a satiety signal comes from microdialysis studies demonstrating that NAc-ACh reaches maximum levels toward the end of a meal and strongly correlates with a decrease in food-seeking and consumption behavior (Mark et al., 1992; Rada et al., 2005; Avena et al., 2008). The physiologic impact of this local rise in ACh on food intake is unclear however, as pharmacological disruptions in NAc-ACh transmission have resulted in conflicting behavioral results (Pratt and Kelley, 2004; Will et al., 2006; Perry et al., 2009; Pratt and Blackstone, 2009). Moreover, selective NAc-CIN lesions both increase food intake over the course of days and decrease the amount consumed after a 24-h fast (Hajnal et al., 2000). In addition, the heterogeneous pre- and postsynaptic distribution of cholinergic receptor subtypes and anatomically distinct ACh populations projecting to the NAc (Woolf and Butcher, 1981; Dautan et al., 2014) have made it difficult to pinpoint the importance of NAc-CIN activity in controlling food intake. These challenges highlight the limitations of traditional pharmacological approaches in linking functionally specialized cholinergic circuits with adaptive behavioral responses. To further characterize the relationship between NAc-CIN activity and satiety-related changes in motivation, the present study used designer receptor (DREADD) technology targeted to NAc-CINs for controlling their activity while mice seek out and consume food. Our results provide further support for NAc-CINs playing an important role in controlling food intake and offer a potential mechanism by which NAc-CINs may act to signal satiety through the inhibition of NAc output.

## Methods

### Experimental animals

All animal procedures were performed in accordance with the UK Animals (Scientific Procedures) Act of 1986. Male heterozygous mice expressing Cre recombinase under the control of the ChAT promoter [ChAT::cre mice (B6N;129S6-Chat^tm2(cre)Lowl^/J); The Jackson Laboratory] were used in the experiments. Mice were housed 2–10 animals per polycarbonate cage and provided *ad libitum* with water and standard lab diet (RM3, Special Diet Services, Essex, UK) in a holding room maintained under a 12-h light cycle (lights off at 7 p.m.) with temperature regulated at 22–24°C and relative humidity kept at 50–55%. Mice were genotyped using PCR from ear notch biopsy. Before behavioral testing, the animals were handled daily for 1 wk. One hour before each behavioral session, the mice were transported to the respective testing area.

### Stereotactic injections

ChAT::cre mice 2–3 mo of age were anaesthetized with isoflurane (5% induction, 1–2% maintenance; Abbott) mixed with oxygen (flow rate 0.8–1.0 l/min) and placed in a stereotactic frame (David Kopf Instruments), the skull was exposed via a small incision, and a small bilateral craniotomy was performed to allow intracranial injections. A stainless steel beveled microinjector was lowered to a coordinate aimed at the NAc (anteroposterior +1.30 mm in relation to bregma, laterally ±0.70 mm in relation to midline, and –4.2 mm and –4.5 mm deep from the skull level). The microinjector was connected to a 1-μl Hamilton glass syringe via polyethylene tubing, and an injection rate of 0.1 μl/min was regulated by a microprocessor-controlled programmable syringe pump (KD Scientific). Starting from the ventral site, each injection site received 150-nl volume of one of the following viruses: AAV2-hSyn-DIO-mCherry, AAV2-hSyn-DIO-rM3D(Gs)-mCherry, AAV2-hSyn-DIO-hM3D(Gq)-mCherry, or AAV2-hSyn-DIO-hM4D(Gi)-mCherry (titer 1–5 × 10^12^ vg/mL, Gene Therapy Center, University of North Carolina School of Medicine, Chapel Hill, NC) followed by a 2-min wait, except for the dorsal site, for which the waiting time was extended to 4 min. For postoperative care, mice received meloxicam (1 mg/kg s.c.; Boehringer Ingelheim), and a recovery period of 5 wks was allowed before behavioral testing.

### Immunohistochemistry

To determine the specificity of receptors expression in CINs, mice were anaesthetized with pentobarbital (500 mg/kg, intra-peritonally (i.p.); Vetoquinol) and transcardially perfused first with 0.1 m PBS followed by 10% neutral-buffered formalin (Sigma-Aldrich). Brains were removed, postfixed overnight at 4°C, and cryoprotected at 4°C with 30% w/v sucrose in PBS until the brains sank and were completely submerged. Coronal sections (30 μm) were cut on a freezing sliding microtome (model 860; American Optical Company). For both mCherry and VAChT immunostaining, sections were washed at room temperature (RT) in 0.1 m PBS, blocked with 1% BSA (Thermo Fisher Scientific) supplemented with 0.3% Triton X-100 (Thermo Fisher Scientific) in 0.1 m PBS. Sections were then incubated overnight at RT in primary antibodies diluted in blocking buffer, washed in PBS, incubated in secondary antibodies for 2 h at RT, washed in PBS, mounted on microscope slides, and coverslipped. Primary antibodies were rabbit anti-mCherry (1:1000, ab167453; Abcam) and guinea pig anti-VAChT (1:500, AB1588; EMD Millipore). Secondary antibodies were donkey anti-rabbit Alexa Fluor 594 (1:1000; Abcam) and goat anti–guinea-pig Alexa Fluor 488 (1:1000; Abcam). Digital images were captured with a Zeiss Axioskop 2 microscope (Zeiss) and QImaging QICAM Fast digital camera (QImaging). Images were merged using ImageJ (National Institutes of Health).

### Electrophysiology

Coronal slices were made >9 wks postinjection. 250-mm-thick slices were cut with a Leica VT 1200S vibratome in ice-cold artificial CSF (ACSF; see below) and allowed to recover for 1 h at 35°C in ACSF before recordings. Patch pipettes were manufactured from borosilicate glass, and their tip resistances were 4–6 MΩ when filled with K-gluconate solution (see below). Whole-cell recordings were conducted at 37°C using an EPC-10 amplifier and Patch-Master software (HEKA Elektronik). Only cells with access resistances of <20 MΩ were used for analysis. Current signals were low-pass filtered at 3 kHz and digitized at 10 kHz. Data were analyzed using Axograph, Patch-Master, and Igor Pro software. Whole-cell recordings were performed at 35°C using an EPC-10 amplifier and Patch-Master software (HEKA Elektronik). ChAT-containing cells were visualized in acute living brain slices using a GFP filter set (Chroma). Clozapine-*N*-oxide (CNO; Sequoia Research Products) was prepared in ACSF and bath-applied at a concentration of 10 µm.

#### Chemicals and solutions

Slice-cutting and recording ACSF was gassed with 95% O_2_ and 5% CO_2_, and contained the following (in mm): NaCl 125, NaHCO_3_ 25, KCl 3, NaH_2_PO_4_ 1.25, CaCl_2_ 1 (cutting)/2 (recording), MgCl_2_ 6 (cutting)/1 (recording), sodium pyruvate 3, and glucose 25 (cutting)/5 (recording). Pipettes were filled with (in mm): potassium gluconate 135, NaCl 7, Hepes 10, Na_2_-ATP 2, Na-GTP 0.3, and MgCl_2_ 2; pH was adjusted to 7.3 with KOH. All chemicals were from Sigma-Aldrich, Tocris, and Abcam.

#### Whole-cell recording protocol

For determining the responsiveness of transduced CINs to CNO, whole-cell recordings were made from identified CINs. Immediately after whole-cell access, current pulses were delivered for identifying signature CIN currents. Typically, CINs were spontaneously active at rest, but in almost all cases, neuronal firing decreased with time. In a few examples, constant positive current was injected for triggering action potentials (Fig. 2*B*). To control for presynaptic changes in excitability, in all cases synaptic antagonists (in μm: CNQX 20, AP5 100, CGP-52432 10, and gabazine 10) were bath-perfused before CNO application. To confirm recording stability, ∼5 min elapsed between whole-cell access and testing for CNO-mediated responsiveness. After 2 min of additional baseline recording, CNO (10 µm) was bath-applied for 3 min, and recordings continued CNO-free for no less than 8 min. A voltage-clamp ramp was delivered before and immediately after CNO termination for determining the change in resting membrane potential (Vm_R_; Fig. 2*B*). Current–voltage (I-V) relationships were obtained by performing voltage-clamp ramps from 0 to −120 mV in 1.5 s.

For determining the impact of CIN activation on medium spiny neuron (MSN) responses, whole-cell recordings were made from putative NAc-MSNs. Immediately after whole-cell access, current steps were delivered for identifying signature MSNs currents. As was the case for whole-cell CIN recordings, ∼5 min elapsed between whole-cell access and testing for CNO-mediated responsiveness. I-V relationships were obtained by performing voltage-clamp ramps from 0 to −120 mV in 1.5 s in voltage clamp immediately before recording baseline activity. Next, while in current-clamp recording mode, current was injected to bring the membrane potential of MSNs close to threshold. After a 2-min baseline period, 10 µM CNO was bath-applied for 3 min to activate Gq-expressing CINs. Changes in Vm_R_ were determined by comparing a MSNs-injected current ramp before (Fig. 7*B*, black trace) recording and one 2 min after (Fig. 7*B*, brown trace) CNO termination. Only stable recordings were included in the dataset.

### Food intake behavior

For habituating animals to the experimental conditions, mice were first gently handled for 1 wk in their home cages, and thereafter placed at the onset of the dark cycle (7 p.m.) on two consecutive days into individual cages for a 2-h food intake measurement. After the habituation, the *ad libitum* diet-fed mice were administered either with CNO (1 mg/kg, i.p., Sequoia) or vehicle (10 mL/kg, i.p., sterile 0.9% saline supplemented with 0.5% DMSO) 30 min before the onset of the dark cycle in their home cages. This i.p. injection of CNO timeframe has been shown to reliably impact virally expressed designer receptors in the CNS (Alexander et al., 2009). At the onset of the dark cycle, mice were transferred to the individual cages with food (standard diet, as above) on the food hopper and water bottle installed, and food intake and body weight were measured. The mice were tested with both CNO and vehicle administered in a counterbalanced order with a 2-d interval between sessions. After the acute food intake experiment, the mice were fasted for 24 h in home cages, and all mice received an injection of CNO (1 mg/kg, i.p.) 30 min before the onset of the dark cycle. At the onset of the dark cycle, the mice were placed into the individual cages with food on the hopper and water bottle installed, and food intake and body weight were measured.

### Operant behavior

#### Apparatus

Experiments were performed in Bussey-Saksida mouse operant touchscreen chambers (Campden Instruments) as presented previously (Mar et al., 2013; Heath et al., 2015). The apparatus has a perforated stainless steel floor and trapezoidal walls bordering the area from a food magazine to a touchscreen (12.1 inches; resolution 800 × 600) equipped with infrared beam arrays at <5 mm from the screen surface to detect nose-poke responses without animals having to apply pressure on the screen for a response to be detected. To guide responding and decrease unintentional touches on the screen, a screen mask made of black acrylic with a row of five 4 × 4-cm openings 1 cm apart from each other and 1.5 cm from the floor level was placed in front of the screen. The white square visual stimulus is presented only in the central opening/location. The apparatus is enclosed in a sound-attenuating chamber with a fan to provide ventilation and mask background noise. The food magazine connects to a pump delivering a reward (Yazoo Strawberry milkshake; Friesland Campina). A LED and a speaker delivered a magazine light and a tone, respectively, at the reward delivery.

#### Touchscreen training

All testing was performed during the light cycle. Before start of experiments, the mice were food restricted to 85–90% of the free-feeding weight with water available *ad libitum* in the home cage throughout. Mice were first habituated to consume the milkshake reward in their home cages for 2 d to avoid hyponeophagia, and to the apparatus for two consecutive days, with all mice consuming 200 μL milkshake that was delivered into the magazine before the session start. To train the mice to associate the stimulus with the reward delivery (20 μL), a 60-min session was started with a presentation of the visual stimulus for 30 s followed by a delivery of a tone (1000 ms, 3 kHz), a magazine light, and a reward. If the visual stimulus was touched three times the quantity of reward was delivered (60 μL). The magazine light was turned off at the reward collection and followed by 5-s intertrial interval (ITI). All animals collected 30 rewards and thus reached the training criterion.

#### Fixed ratio training

After the initial training, the mice were trained for fixed ratio (FR) performance with the following parameters: stimulus removal for 500 ms, session length of 60 min, reward volume of 20 μL, ITI of 4.5 s, and tone of 10 ms at 10 kHz. Mice were first trained to FR1 (one screen touch for reinforcer delivery) and FR3, then continued to FR5. Animals reached the criterion when 30 trials were completed during a session.

#### FR5 uncapped probe

Animals were administered with CNO (1 mg/kg, i.p.) or vehicle 30 min before placing them into the chambers and tested for FR5 performance with no trial limit in 60-min sessions as in FR training. The mice were tested with both CNO and vehicle administered in a counterbalanced order with a 3-d interval with a break day and a training session in between the test sessions.

#### Progressive ratio

Next, mice were tested on a progressive ratio 4 (PR4) schedule that required incremental increases in the number of touches required for reinforcer delivery, as previously described (Heath et al., 2015). Sessions were terminated after either 5 min of inactivity or after 60 min had elapsed. The mice were tested with CNO (1 mg/kg, i.p.) or vehicle administered in a counterbalanced order with a single baseline nondrug day between each drug test session.

#### Data analysis and statistics

All touchscreen testing data were automatically stored in a database within ABET II touch testing software. The total response time was defined as the amount of time between the first and last touch of a single trial and converted to rate for both FR and PR sessions. Individual sessions of FR response rate data were binned in blocks of 20 trials and fitted with the parabolic function *y* = *b* * *x*^2^ + *a* for stabilizing the variance and normalizing the distribution. Individual sessions of PR response rate data were fitted with the negative exponential function *y* = *a^–b^* ^*^^n^ as per previously reported analysis (Bailey et al., 2016). These data were analyzed using repeated-measures ANOVA with trial bin and CNO/vehicle condition as within-subject factors. Post-reinforcement pause (PRP) was defined as the time elapsed between the removal of the head from the reward magazine and the emission of the first touch of the next trial. These data were binned in blocks of 50 trials for FR sessions. Statistical analysis of this measure was conducted using linear mixed models, as this class of statistical modeling tolerates incompleteness, and animals cumulatively stopped responding throughout the session. All statistical testing was conducted with a significance level of *p* < 0.05.

## Results

### Designer receptors are expressed in NAc-CINS and in sufficient quantities for modulating their excitability

**Figure 1.**
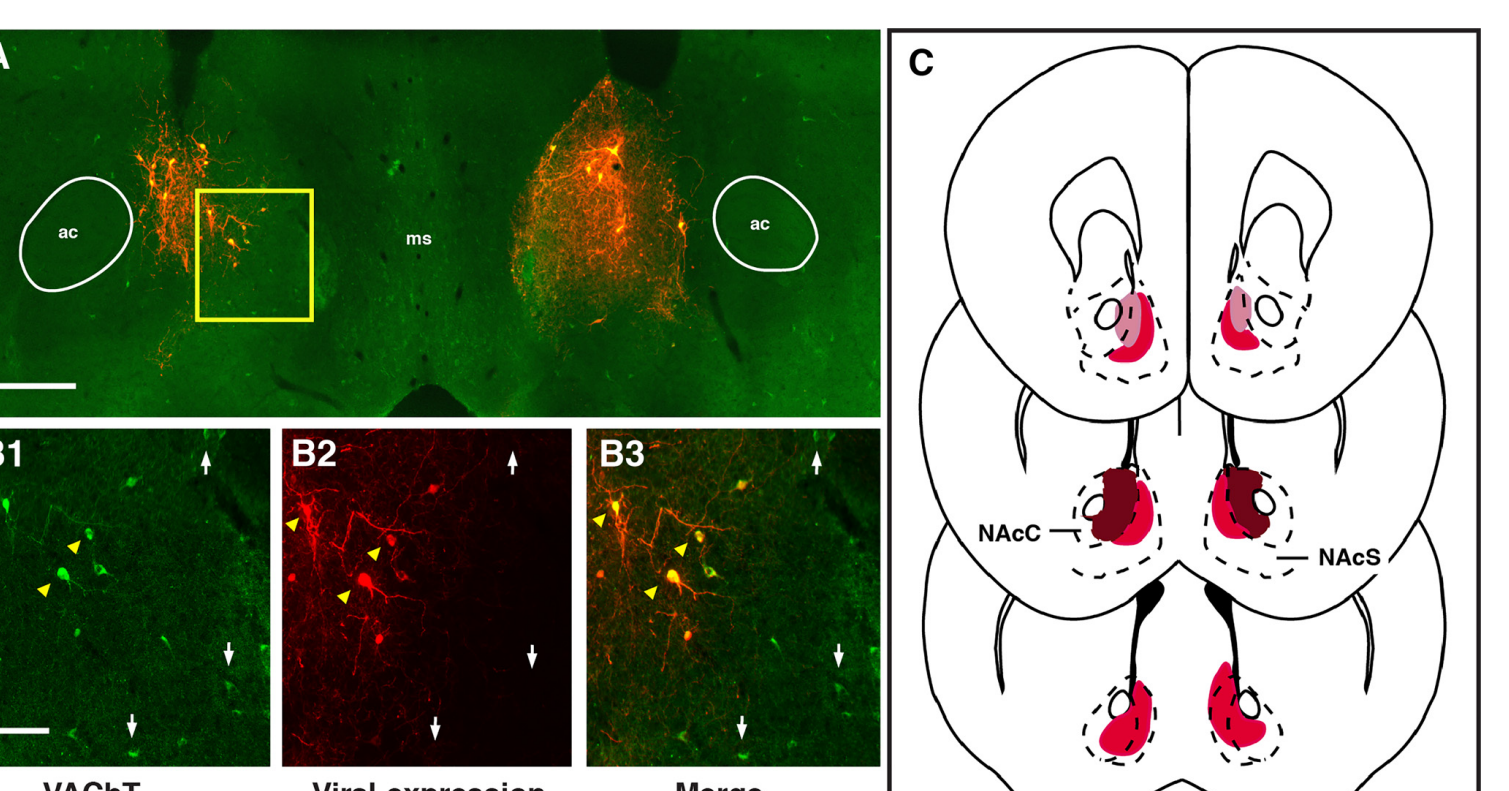
Selective expression of viral constructs in NAc cholinergic interneurons. Viral targeting of NAc-CINs in ChAT-cre mice resulted in selective expression of MCY (***B2***) in neurons expressing the vesicular ACh transporter VAChT (***B1***, ***B3***) in the medial core and shell region of the NAc (*n* = 10; ***A***, ***C***). ***C***, Darker shading corresponds to the qualitative greater density of the dendritic and somatic extent of ACh-CIN labeling. ac, anterior commissure; ms, medial septum; NAcC, nucleus accumbens core; NAcS, nucleus accumbens shell. White arrows, VAChT-expressing neurons; yellow arrowheads, neurons expressing both VAChT and MCY. Scale bars: ***A***, 100 μm; ***B***, 0.5 mm.

**Figure 2.**
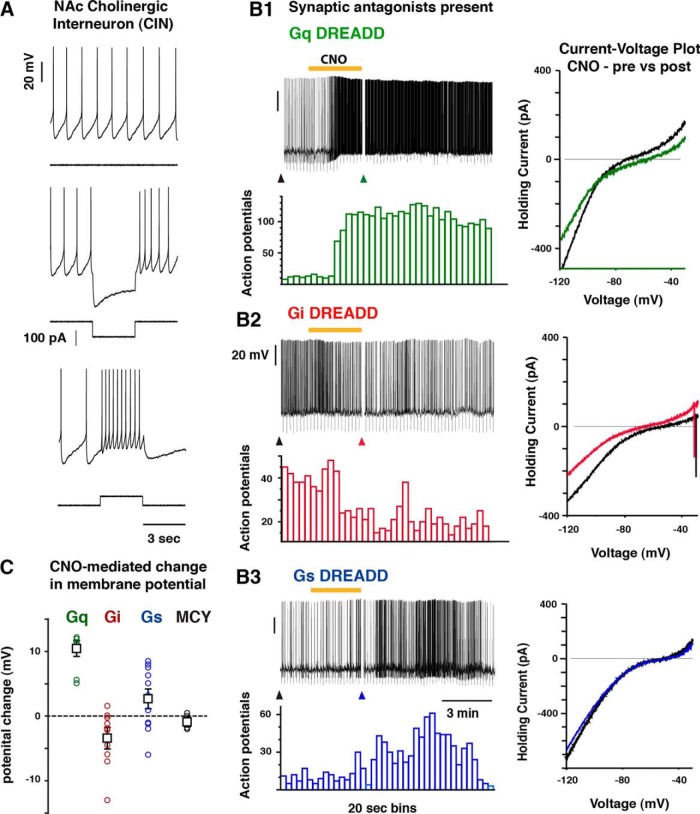
Designer receptor expression was sufficient for modulating CIN excitability *in vitro*. ***A***, Recorded neurons were regular spiking and mostly spontaneously active at rest and showed signature CIN intrinsic currents. ***B***, Bath application of 10 μM CNO reliably depolarized the membrane potential of neurons expression Gq-coupled (*n* = 10; ***B1***, ***C***) and Gs-coupled (*n* = 11; ***B3***, ***C***) designer receptors, whereas neurons expressing Gi-coupled (*n* = 9) receptors were inhibited (***B2***). Black arrowhead (left), baseline current ramp injection; colored arrow (right), post-CNO current ramp injection. ***C***, Results summary for CNO-mediated response from left to right in Gq-expressing (green), Gi-expressing (red), Gs-expressing (blue), and MCY-only (black; *n* = 6) CINs plotted as the change in membrane potential. Synaptic antagonists (in μm) = CNQX 20, AP5 100, CGP-52432 10, gabazine 10.

For effectively manipulating NAc-CIN activity *in vivo*, we used a Cre-recombinase approach to express DREADDs in ChAT-cre^+^ transgenic mice. Viral constructs that contained mCherry only (MCY), Gq, Gs, or Gi-coupled DREADDs were bilaterally injected in the medial division of the NAc core and shell. Immunocytochemical analysis revealed that viral expression in CINs was specific, since MCY-expressing neurons were seen to be immunopositive for the vesicular ACh transporter VAChT with high specificity (Fig. 1*A*, *B*). Consistent with previous studies (Bolam et al., 1984; Phelps et al., 1985; Contant et al., 1996; Zhou et al., 2002), the extent of their dendritic and axonal processes was limited to the NAc core and shell and did not extend outside the NAc (Fig. 1*A*, *C*). To determine whether the DREADD-specific ligand CNO was indeed sufficient to modulate CIN activity, we performed whole-cell recordings from identified transduced cells in acute brain slices. Recorded neurons were regular spiking, mostly spontaneously active at rest, and showed signature CIN intrinsic currents (Fig. 2*A*). As expected, in the presence of 10 µm CNO, Gq-expressing CINs consistently showed a depolarization (Fig. 2*B1*, *C*; Vm_R_: *n* = 10; control, –60.7 ± 3.1 mV; CNO, –49.9 ± 3.4 mV; paired *t* test, *p* = 0.0007), whereas Gi-expressing CINs were hyperpolarized (Vm_R_: *n* = 9; control, –63.8 ± 3.8 mV; CNO, –67.2 ± 3.9 mV; paired *t* test, *p* = 0.02) with bath application of CNO (Fig. 2*B2*, *C*). CNO-evoked responses in Gs CINs were significantly depolarizing (Vm_R_: *n* = 11; control, –55.4 ± 2.7 mV; CNO, –52.8 ± 2.6 mV; paired *t* test, *p* = 0.04), but the responses were smaller in magnitude than those seen in Gq-expressing CINs (change in mV: Gq = 10, 10.8 ± 1.0 mV; Gs = 11, 2.5 ± 1.5 mV; unpaired *t* test, *p* < 0.0001; Fig. 2*C*). In contrast, there was no change in the membrane potential of MCY-expressing CINs after CNO application (Vm_R_: *n* = 6; control, –62.3 ± 4.8 mV; CNO, –63.8 ± 4.8 mV; paired *t* test, *p* = 0.13). Comparisons of CIN input resistance before and after CNO delivery revealed a significant decrease in Gi-expressing CINs (*n* = 9: Control, 329 ± 28 MΩ; CNO, 282 ± 24 MΩ; paired *t* test, *p* = 0.02) whereas neither Gq- nor Gs-expressing CINs showed any change in response to CNO (Gq, *n* = 10; control, 341 ± 22 MΩ; CNO, 359 ± 43 MΩ; paired *t* test, *p* = 0.56: Gs, *n* = 9; control, 327 ± 62 MΩ; CNO, 318 ± 55 MΩ; paired *t* test, *p* = 0.564). Together, these results provide evidence that Gq-, Gi-, and Gs-linked designer receptors were selectively expressed in NAc-CINs and in sufficient quantities for modulating their excitability.

### CIN activity modulation does not change normal feeding behavior

**Figure 3.**
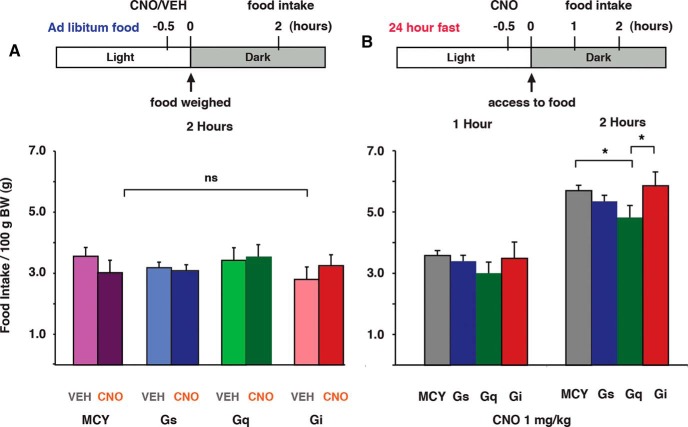
Impact of CIN activity modulation on food intake after free food access and a 24-h fast. ***A***, MCY, Gs, Gq, and Gi NAc-CIN–expressing mice with *ad libitum* food access were given an injection of either VEH or CNO 30 min before the dark cycle (active phase), and food consumption was measured 150 min later. ***B***, CNO injections 30 min before the active phase in mice fasted for 24 h resulted in a significant reduction in food intake for Gq-expressing compared with both MCY- and Gi-expressing mice at 2 h after reintroducing home-cage food. Gq, *n* = 10; Gs, *n* = 14; Gi, *n* = 10; MCY, *n* = 15. *, *p* < 0.05.

Previous pharmacological research investigating the cholinergic regulation of NAc-dependent feeding behavior has resulted in conflicting results, with reports of both ACh receptor-specific increases and decreases in food intake that have been attributed to NAc-CINs (Hoebel et al., 2007; Baldo et al., 2013). However, CIN communication is not restricted to ACh release, as CINs are known to excite postsynaptic targets by releasing glutamate (Higley et al., 2011; Nelson et al., 2014a, b). Moreover, recent anatomic data has shown that mesopontine brainstem ACh neurons also project to the NAc (Dautan et al., 2014), indicating that cholinergic regulation of NAc processes is not restricted to CIN activity. As such, the relationship between NAc-CIN activity and food intake remains unresolved. Using a designer-receptor approach *in vivo*, we investigated how modifying the excitability of NAc-CINs affected feeding behavior. To determine the impact of CIN activity on daily home-cage feeding bouts, mice expressing Gq-linked (*n* = 10), Gs-linked (*n* = 14), and Gi-linked (*n* = 10) linked DREADDs and MCY only (*n* = 15) in NAc-CINs were injected with either CNO or vehicle (VEH) 30 min before the start of the active phase, when mice normally show a significant increase in food intake. For all conditions, VEH and CNO injections were counterbalanced over two testing sessions that were separated by 2 days. Regardless of whether they received CNO or VEH injection, mice ate an equal amount of home-cage diet 2 hours after the start of the active phase (two-way ANOVA: group effect, *F*_(3,90)_ = 1.00, *p* = 0.40; drug effect, *F*_(1,90)_ = 0.003, *p* = 0.96; interaction, *F*_(3,90)_ = 1.28, *p* = 0.29; Fig. 3*A*). In addition to demonstrating that CNO in control conditions (MCY group) has no impact on normal eating behavior, these results provide evidence that changes in CIN activity do not adversely affect food intake by inducing taste aversion, as increases in NAc-ACh activity have been previously shown to do (Taylor et al., 2011).

### Under states of high motivation, CIN modulation differentially affects food intake

In the preceding experiment, the motivation to consume food was relatively low, since animals had free access to homecage food in their normal feeding environment. Previous studies have shown CINs to be important in reward-based behavior when the stimuli used are strongly reinforcing, such as drugs of abuse and natural rewards (Hajnal et al., 2000; Hikida et al., 2001, 2003). To test for the involvement of NAc-CIN under heightened levels of motivation, we increased the incentive value of the home-cage food by fasting mice for 24 hours before reintroducing food 30 min after CNO injections. Previous studies have demonstrated that when fasted on multiple occasions, mice increase their activity in anticipation for food (Mieda et al., 2004; Storch and Weitz, 2009). To avoid potential confounds from altered behavior induced by multiple fasting events, a single fasting event was imposed, therefore allowing for a between-subject comparison. At 1 hour after the reintroduction of home-cage food, differences in food consumption between MCY and Gq-expressing mice began to emerge but were not statistically different (two-way ANOVA; between groups, *F*_(3,95)_ = 1.44, *p* > 0.05; within-group, *F*_(1,95)_ = 3.08, *p* > 0.05; Fig. 3*B*). At 2 hours, however, Gq mice consumed significantly less than MCY and Gi mice (MCY, 5.7 ± 0.2; Gq, 4.8 ± 0.6; Gi, 5.9 ± 0.4 g/100 g body weight; one-way ANOVA, group effect *F*_(3,44)_ = 3.70, *p* = 0.01; Bonferroni *post hoc* test; MCY vs. Gq *p* < 0.05, Gi vs. Gq *p* < 0.05; Fig. 3*B*), suggesting that increases in NAc-CIN activity, when animals are motivated by way of a 24-h fast, can act to reduce food consumption.

**Figure 4.**
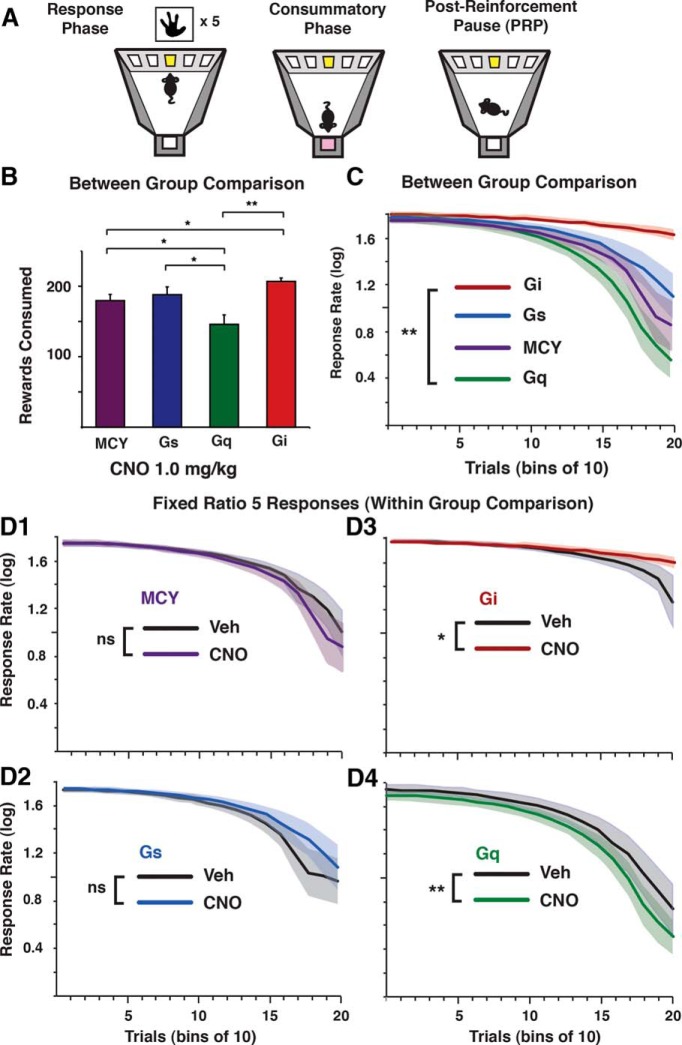
Differential modulation of CIN excitability impacts food-seeking behavior in a time-dependent manner. ***A***, Mice were trained on a FR5 schedule of reinforcement for a palatable food reward. ***B***, Summary of trials completed after CNO injection in DREADD-expressing and MCY mice. ***C***, Between-group comparison of response rate over time. ***D***, Within-group comparisons (VEH vs. CNO) of response rate over time for all groups. MCY (D1), *n* = 12; Gs (D2), *n* = 14; Gi (D3), *n* = 10; Gq (D4), *n* = 10. *, *p* < 0.05; **, *p* < 0.01.

These results are in agreement with past research relating increased NAc-ACh to reduced seeking behavior for natural rewards (Rada et al., 2005; Avena et al., 2006) and for drugs of abuse (Hikida et al., 2001, 2003; Zhou et al., 2007). Based on such findings, it has been proposed that NAc-ACh can act to promote satiety signaling by reducing the incentive value of rewarding stimuli. To shed light on the interaction between hunger, food reward, and satiety signaling, we trained food-restricted mice to perform an operant task with a FR schedule of reinforcement for receiving a palatable food reward (Fig. 4*A*). The motivating nature of the stimulus and the spaced feeding bouts provided by a FR regimen allowed us to measure changes in response rate that may reflect the cumulative decrease in food intake that underlies satiety signaling. Animals were trained to touch an illuminated touchscreen location five times (FR5) to receive 20 μL of a palatable food reward delivered to a well opposite the touchscreen. Consistent with our results demonstrating a relationship between CIN activity and a reduction in food intake in fasted mice, activating NAc-CINs with Gq- and Gi-linked DREADDs resulted in an respective decrease and increase in total trials completed, compared with MCY animals (MCY, 180 ± 6; Gq, 150 ± 13; Gi, 207 ± 5; Gs, 188 ± 11; one-way ANOVA: group effect *F*_(3,44)_ = 5.80, *p* = 0.002; Bonferroni *post hoc* test; MCY vs. Gq *p* = 0.04, Gs vs. Gq *p* < 0.03, Gi vs. Gq *p* = 0.009, MCY vs. Gi *p* = 0.02; Fig. 4*B*).

**Figure 5.**
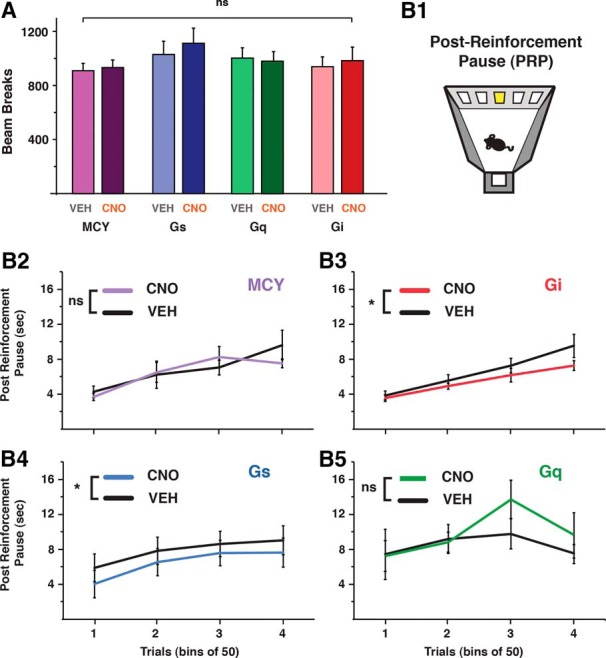
Modulation of CINs impacts measure of satiety. ***A***, In the FR5 operant task, all groups in VEH and CNO conditions showed similar total locomotor activity. ***B***, Within-subject comparisons for the time taken, after reward consumption, for all groups to start a new trial (PRP). MCY (B2), *n* = 12; Gi (B3), *n* = 10; Gs (B4), *n* = 14; Gq (B5), *n* = 10. *, *p* < 0.05.

To further characterize the impact of CINs on food-seeking behavior, we next measured changes in response rate between CNO-injected and control mice throughout the entire 1-hour session (Fig. 4). Despite equal initial rates, Gq (*n* = 10) animals responded significantly less than Gi (*n* = 10) animals toward the end of the session (one-way ANOVA: group effect *F*_(3,42)_ = 3.39, *p* = 0.02; Bonferroni *post hoc* test; Gi vs. Gq *p* = 0.01; Fig. 4*C*), whereas the VEH-treated groups were not significantly different from one another (one-way ANOVA: group effect *F*_(3,42)_ = 1.62, *p* = 0.20; data not shown). Within-subject comparisons (VEH and CNO sessions) for each group revealed a significant interaction between trial and injection type (CNO vs. VEH) where Gq activation led to a reduction in responding (one-way ANOVA with repeated measures, group effect *F*_(1,9)_ = 5.07, *p* > 0.05; interaction, bin * treatment, *F*_(19,171)_ = 1.96, *p* = 0.01; Fig. 4*D4*), whereas Gi-mediated inhibition of NAc-CINs resulted in an elevated level of food seeking compared with control conditions (one-way ANOVA with repeated measures: group effect *F*_(1,9)_ = 3.22, *p* > 0.05; interaction, bin * treatment, *F*_(19,171)_ = 2.65, *p* < 0.001; Fig. 4*D3*). In contrast, neither MCY (*n* = 12) nor Gs (*n* = 14) animals treated with CNO showed altered food-seeking behavior compared with treatment with VEH (one-way ANOVA with repeated measures: Gs group effect *F*_(1,11_) = 0.58, *p* > 0.05, interaction, bin * treatment, *F*_(19,209)_ = 0.68, *p* > 0.05; MCY group effect *F*_(1,13_) = 0.78, *p* > 0.05, interaction, bin * treatment, *F*_(19,247)_ = 1.36, *p* > 0.05; Figs. 4*D1*, *D2*). For all groups, mice injected with either CNO or VEH were equally active throughout the session (two-way ANOVA: group effect, *F*_(3,84)_ = 1.54, *p* = 0.21; drug effect, *F*_(1,84)_ = 0.31, *p* = 0.58; interaction, *F*_(3,84)_ = 0.15, *p* = 0.93), suggesting that there was no overt behavioral deficit in responding (Fig. 5*A*).

We next measured the time after reward consumption before animals voluntarily begin the next trial (Fig. 5*B1*). This delay, known as the post-reinforcement pause (PRP) has been shown to increase with food consumption and is thought to reflect the decrease in motivation for food due to satiety (Sidman and Stebbins, 1954; Patrikiou and Keehn, 1964; Felton and Lyon, 1966). Consistent with the notion that changes in CIN activity can affect motivation for food, Gi-expressing mice injected with CNO showed a significant decrease in PRP magnitude compared with control mice (one-way ANOVA: Gi main effect by treatment *F*_(1,67)_ = 6.16, *p* = 0.01; interaction, bin * treatment *F*_(3,63)_ = 1.17, *p* > 0.05; Fig. 5*B3*). Gq animals treated with CNO, however, did not show an increase in PRP compared with control conditions (one-way ANOVA: Gq main effect by treatment *F*_(1,45_) = 3.24, *p* = 0.08; interaction, bin * treatment *F*_(3,45)_ = 1.81, *p* > 0.05; Fig. 5*B5*). Similarly, CNO-treated MCY mice showed no difference compared with control conditions (one-way ANOVA: MCY, main effect by treatment *F*_(1,81)_ = 0.34, *p* > 0.05; interaction, bin * treatment, *F*_(3,81)_ = 0.66, *p* > 0.05; Fig. 5*B2*). Surprisingly, Gs mice showed a significant difference in control and CNO conditions (main effect by treatment *F*_(1,79)_ = 5.01, *p* < 0.05; interaction, bin * treatment *F*_(3,79)_ = 0.06, *p* > 0.05).

### Stimulus incentive value that is largely independent of satiety signaling is unaffected by CIN modulation

**Figure 6.**
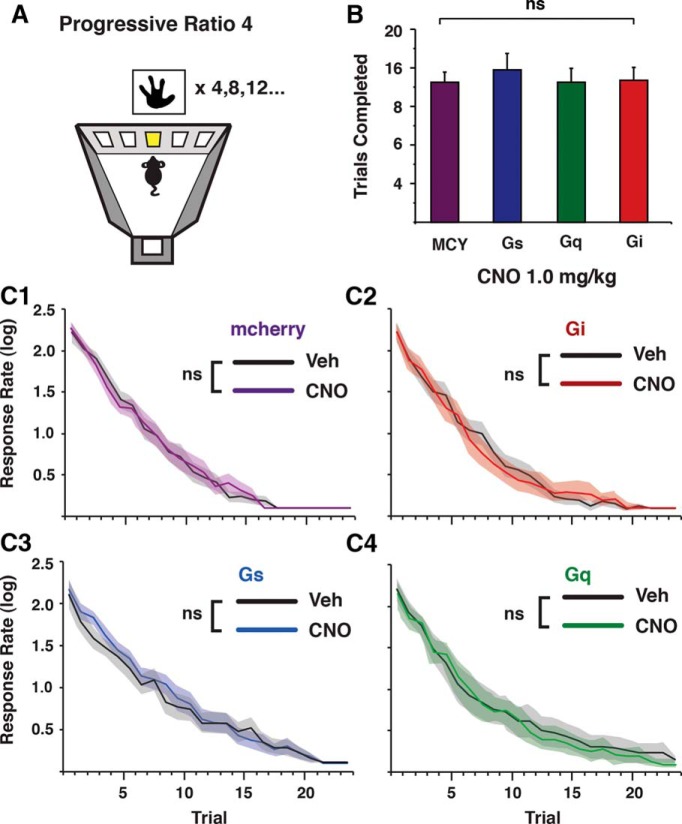
Reinforcer strength largely independent of satiety signaling is unaffected by CIN modulation. ***A***, Mice were trained on a PR schedule of reinforcement in which correct touches for a palatable food reward consecutively increased by four. ***B***, All groups completed an equal amount of trials, thus consuming a similar amount of food reward. ***C***, Within group comparisons (VEH vs. CNO) of response rate over time or all groups. MCY (C1), *n* = 12; Gi (C2), *n* = 10; Gs (C3), *n* = 14; Gq (C4), *n* = 10.

By demonstrating that changes in CIN excitability can have opposing effects on food intake, these results indicate that CINs play an active role in regulating motivation. In is unclear, however, whether these CIN-induced differences in motivation were due to changes in the innate reinforcer strength of the stimuli that were unrelated to prior food consumption. To test this, mice were trained on a PR schedule of reinforcement in which, after successful trial completion, the correct touches necessary for food delivery incrementally increased by four (Fig. 6*A*). The PR task is well known to gauge reinforcer strength (Hodos, 1961; Eagle et al., 1999) that is mostly independent of satiety signaling. The increased behavioral demand imposed by this schedule of reinforcement results in significantly less food being consumed compared with a FR5 regimen. In the current study, PR4 and FR5 control mice consumed on average 14.4 ± 1.1 (≈288 μL) and 169.5 ± 7.4 (≈3.4 mL) rewards, respectively. The small amount of food consumed in the PR4 task allowed us to determine whether the time-dependent changes in response rates could be attributed to adjustments in reinforcer strength that is largely independent of satiety signaling. Both between-subject (Fig. 6*B*) and within-subject (Fig. 6*C*) analyses revealed that modulating CIN activity while animals sought food rewards in a PR4 task had no significant effect on trials completed (one-way ANOVA: group effect *F*(3,44) = 0.80, *p* > 0.05; Fig. 6*B*) or on response rates (within group comparisons one-way ANOVA with repeated measures, group effect: MCY (*n* = 12); *F*_(1,13)_ = 0.52, *p* > 0.05; interaction, bin * treatment, *F*_(19,247)_ = 0.75, *p* > 0.05. Gs (*n* = 14): *F*_(1,13)_ = 0.39, *p* > 0.05; interaction, bin * treatment, *F*_(19,247)_ = 0.71, *p* > 0.05. Gq (*n* = 10): *F*_(1,9)_ = 0.05, *p* > 0.05; interaction, bin * treatment, *F*_(19,171)_ = 0.42, *p* > 0.05. Gi (*n* = 10): *F*_(1,13)_ = 0.16, *p* > 0.05; interaction, bin * treatment, *F*_(19,247)_ = 0.97, *p* > 0.05; Fig. 6*C*), supporting the viewpoint that CIN activity does not change the overall incentive value of the rewarding stimuli.

### Chemo-genetic activation of CINs inhibits accumbal output neurons through nicotinic and GABAergic receptor activation

**Figure 7.**
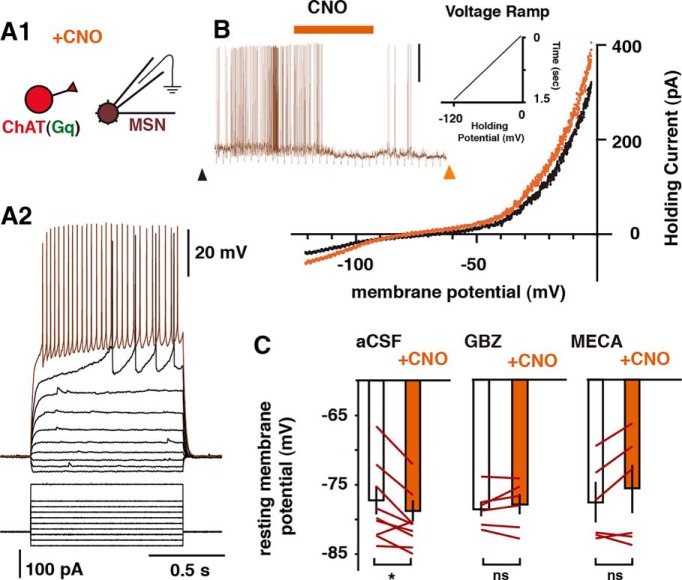
Designer receptor activation of CINs inhibits MSNs through GABA_A_ and nicotinic receptor activation. ***A1***, Setup schematic: *in vitro* whole-cell recordings from MSNs while bath-applying CNO onto Gq-expressing NAc-CINs. ***A2***, Example of a typical MSN recording showing changes in membrane potential in response to current injections. ***B***, Example trace showing a reduction of MSN activity after bath application of CNO and corresponding I-V relationship taken before (black trace, arrowhead) and after (orange trace, arrowhead) CNO application. In this example, the membrane potential was held at above threshold with current injections through the intracellular electrode. ***C***, Group summary of CNO-evoked changes in membrane potential in control conditions (*n* = 9; left), when pretreated with the GABA_A_ receptor antagonist gabazine (GBZ, 10 μM; *n* = 6; middle), or with the nicotinic receptor antagonist mecamylamine (MECA, 10 μM; *n* = 6; right).

It has previously been shown that NAc-CINs can effectively inhibit NAc output MSNs (Witten et al., 2010; Nelson et al., 2014a). In these studies, activating NAc-CINs optogenetically with millisecond precision while recording responses in MSNs showed that the CIN-evoked inhibition was likely due to an increase in GABA_A_ receptor activation (De Rover et al., 2002; Nelson et al., 2014a). DREADD approaches lack such clear temporal relationships, since activity is manipulated through G-protein–coupled second messenger pathways. We thus tested *in vitro* whether activating CINs using Gq DREADDs can similarly inhibit MSNs (Fig. 7*A*). Bath application of 10 μm CNO in acute brain slices expressing Gq DREADDs in CINs resulted in a significant hyperpolarization of MSNs (*n* = 9: control, –76.2 ± 2.1 mV; CNO, –78.6 ± 1.5 mV; paired *t* test, *p* = 0.03; Fig. 7*B*, *C*, left) that was dependent on GABA_A_ receptor activation (pretreatment with 10 μM gabazine, *n* = 6: control, –78.9 ± 1.0 mV; CNO, –77.1 ± 2.1 mV; paired *t* test, *p* = 0.46; Fig. 7*C*, middle). In addition, these changes in membrane potential were similarly prevented by pretreating the tissue with the nonspecific nicotinic receptor antagonist mecamylamine (10 μM; *n* = 6: control, –76.6 ± 3.2 mV; CNO, –74.9 ± 3.9 mV; paired *t* test, *p* = 0.15; Fig. 7*C*, right), a result consistent with recent work showing that CIN activation in the dorsal striatum can drive GABA release from local interneurons that decreases MSN activity (Nelson et al., 2014a, b). These results suggest that Gq-mediated activation of NAc-CINs may act to release ACh that then influences local NAc networks.

## Discussion

The present study demonstrates that modulation of NAc-CIN activity is sufficient to bring about changes in food-motivated behaviors. Through DREADD-mediated bidirectional control of CIN activity, we show that, when motivated by food restriction, increasing CIN activity led to a reduction in palatable food consumption, whereas reducing CIN excitability enhanced food intake. These activity-dependent changes developed late in the session and were unlikely to be driven by changes in the innate reinforcer strength, since CIN manipulation did not affect operant responses to the reinforcer when food consumption was minimal. These results support the idea that CIN modulation was instead affecting the cumulative change in motivation underlying satiety signaling. Consistent with past findings, we found that activating CINs *in vitro* led to an inhibition of NAc output neurons that was dependent on both GABA_A_ and nicotinic receptor activation, suggesting that NAc-CINs can act to dampen MSN activity through a feedforward activation of local GABAergic circuits.

These bidirectional changes in food-seeking behavior were induced through Gq- or Gi-linked modified muscarinic second messenger systems. Because muscarinic M3 receptors are found in low levels in the striatum (Yasuda et al., 1993), these effects were unlikely caused by disrupted M3 receptor signaling but instead were likely caused by increases in CIN activity (Fig. 2*B1*, *C*). It is conceivable, however, that Gi-mediated increases in responding were attributed to disruptions in M2/4 second messenger signaling, as activating M2/4 autoreceptors on CINs can reduce Ca^2+^ influx (Yan and Surmeier, 1996). Occluding this signaling pathway would potentially disrupt this Ca^2+^ modulation, resulting in elevated excitability of CIN axon terminals. We predict that such a scenario would result in enhanced transmission and would therefore resemble the end effect of Gq activation. In contrast to Gq-activated CINs, inhibiting CINs through Gi-DREADDs increased food-seeking behavior. These results thus support the interpretation that our manipulations had a bidirectional impact on CIN excitability that resulted in opposing influences on food-seeking behavior. Similar to previous studies, many of these responses lasted well after CNO washout (Alexander et al., 2009). Notably, despite Gs-expressing CINs showing CNO-mediated changes in activity *in vitro,* these changes in excitability were insufficient to affect behavior.

### NAc cholinergic control of food intake

Our results are consistent with previous studies demonstrating a link between NAc-ACh transmission and decreases in food-directed motivation. Past studies have shown that toward the end of a meal there is a local rise in NAc-ACh that strongly correlates with reductions in food intake (Mark et al., 1992), the time course of which can be extended in conditions where meal duration is prolonged (Rada et al., 2005; Avena et al., 2008). Moreover, mimicking such rises (as when ACh breakdown is prevented) results in a significant decrease in food intake (Mark et al., 2011). Similarly, increasing endogenous ACh activity has been shown to reduce approach behaviors toward drugs of abuse (De la Garza and Johanson, 1982; Hikida et al., 2003), whereas eliminating NAc-CINs with neurotoxins reduced such addiction-like behaviors (Hikida et al., 2003). Together, these results support the premise that NAc-CINs can act to reduce the reinforcing effects of rewarding stimuli and therefore influence reward-based behavior.

Despite much experimental support for NAc-CIN activity acting as a motivational stop signal, there has been discrepant pharmacological data demonstrating a decrease, increase, or no impact on appetitive responding after inactivation of local NAc muscarinic receptors (Mark et al., 2006; Will et al., 2006; Perry et al., 2009; Pratt and Blackstone, 2009; Nunes et al., 2013). The significance of CINs for food-seeking behavior has further been confounded by recent anatomic work demonstrating a significant brainstem cholinergic input to the NAc (Dautan et al., 2014), thus revealing alternative cholinergic influences on NAc function. Moreover, in addition to ACh release, central cholinergic neurons have been shown to affect postsynaptic targets through glutamatergic mechanisms (Witten et al., 2010; English et al., 2012; Nelson et al., 2014a, b). Despite our results demonstrating a bidirectional influence of NAc-CINs on food-seeking behavior, the interplay between these different circuits and their synaptic influences on motivation remains unresolved.

### Dual role for CINs in NAc-mediated motivation

Recent work has demonstrated that optogenetic inactivation of NAc-CINs reduces conditioned cocaine place preference (Witten et al., 2010), indicating that CINs, in seeming contrast to our results, are involved in promoting appetitive conditioning. Consistent with this, *in vivo* recordings made from putative CINs in the ventral medial striatum showed that during a food-reward learning task, CINs increased their firing rate specifically at the end of rewarded trials, thus demonstrating that CINs may be encoding for reinforcing outcomes (Atallah et al., 2014). Interestingly, these learning-related responses were not seen when animals were engaged in a well-learned task, suggesting that CIN activity may act to reinforce behavior in the early stages of acquisition. This idea is consistent with the viewpoint that fine-tuning CIN activity can regulate local NAc dopaminergic transmission (Cachope et al., 2012; Threlfell et al., 2012) for supporting reward-based learning (Brown et al., 2012). Notably, the modification of CIN excitability in the present study occurred in the context of a familiar home cage and on well-trained operant tasks (FR5 and PR4). Our results may support a differential role for NAc-CINs between initial reinforcement learning and incentive updating in well-learned conditions, as is the case with satiety signaling. Consistent with this view, NAc-ACh transmission can act to suppress the reinstatement of heroin-seeking on a well-learned operant task (Zhou et al., 2007). More recently, Lee et al. (2016) further implicated CINs in updating well-learned reinforced behavior by showing that NAc-CIN manipulation can modulate extinction learning. The wide range of motivational changes observed after NAc-CIN manipulation suggests that this cellular population likely integrates signals from multiple sources to modulate at different times motivational output.

### Inhibitory CIN impact on NAc output

From a circuit-level perspective, the CIN-mediated decrease in food-seeking behavior may result from an overall increase in inhibitory tone onto NAc output neurons. Previous studies have shown that optogenetically activating CINs can effectively inhibit MSNs (Witten et al., 2010). Consistent with the known connectivity of CINs residing in the dorsal striatum (English et al., 2012; Nelson et al., 2014a; Faust et al., 2015), our results indicate that inhibitory GABA responses evoked by CNO onto Gq-expressing NAc-CINs were dependent on nicotinic receptor activation and were likely due to the feedforward activation of local interneurons that inhibit NAc-MSNs. Different populations of MSNs that can be broadly classified by their neuropeptide and dopamine (D1 and D2) receptor expression have been shown to differentially affect the rewarding aspects of cocaine. Whereas D1-type MSN activation can drive cocaine-mediated learning, D2-type MSN activation has the opposite effect (Lobo et al., 2010). More recently, it has been shown that in mice activating D1- as well as D2-expressing NAc-MSNs can increase motivation for food rewards (Soares-Cunha et al., 2016). Interestingly, devaluing the food reward by allowing mice free access to food before the behavioral task completely disrupted the enhanced motivation after D2-MSN stimulation. Moreover, optical inhibition of D2-MSNs diminished operant responding for food. These results are consistent with our current findings demonstrating that DREADD-activated CINs can reduce food-seeking behavior while also decreasing the excitability of NAc-MSNs. Whether or not these synaptic effects were preferentially on D1- or D2-expressing MSNs was not investigated in the current study. Dopaminergic inputs to spatially distinct CINs have been shown to differentially impact their activity (Chuhma et al., 2014), but whether specialized differences in microcircuit connectivity between NAc-CINs and MSNs exist remains unclear.

Our results support a role for NAc-CINs in bidirectionally influencing satiety. One potential mechanism by which CIN activity can translate into reduced food-seeking behavior is via an indirect inhibition of NAc-MSNs. The afferents that drive CIN activity remain unclear, however. Potential satiety-related sources that are positioned to impact CINs include hypothalamic energy-sensing circuits (Sternson, 2013) and amygdala neurons known to encode for reappraising stimulus value (Morrison and Salzman, 2010). Interestingly, recent work has revealed that CINs express insulin receptors whose activation excites CINs (Stouffer et al., 2015), thus demonstrating an additional influence on CINs with the appropriate time course for progressively driving satiety. A more complete understanding of the various circuits that impact CINs will help reveal the various influences on CINs that contribute to their regulation over the drive to eat.

## Synthesis

Reviewing Editor: Lorna Role, Stony Brook Decisions are customarily a result of the Reviewing Editor and the peer reviewers coming together and discussing their recommendations until a consensus is reached. When revisions are invited, a fact-based synthesis statement explaining their decision and outlining what is needed to prepare a revision will be listed below.

Summary

The authors of this study use cell type-specific manipulations of neuronal activity selectively in Nac-CINs to show that CIN signaling directly affects aspects of feeding and satiety. First, they demonstrate in vitro that DREADD receptor expression and activation can be used to differentially modulate firing properties of Nac-CINs. They then go on to show that manipulating their activity in vivo influences feeding and reward associated behavior that can be linked to satiety.

Although the study lacks mechanistic and/or circuit-level insight as to the downstream effects of manipulating Nac-> CINs, the authors did respond effectively to prior reviews, and indeed provide the novel observation that Nac-CINs directly gate striatal neuron circuitry towards feeding behaviors. Although additional study is needed to better understand the mechanistic details underlying these phenomena, this study provides an important in-road to these questions with state-of-the-art techniques.

After conferring on the review of this manuscript we identified several issues that must be addressed. First, the reviewers have identified some additional controls and experimental details that require attention/ discussion. Second, there are aspects of the methodological approaches used that should be better delineated. Finally, we ask that the authors employ the more informative data display of scatter +/- box plots.

1. Additional controls needed:

----Additional Controls and queries relevant to Fig 2,

a. important conclusions derived from CIN patch recordings should be replicated in the cell-attached mode to ensure that CIN tonic firing does not run down.

b. There should be controls recording from the virus that only expresses mCherry: this will show if there are effects of the drug alone in the absence of DREADD receptors. This is a strong possibility because some studies indicate there are CNO metabolites. The responses due to DREADD should be absent, but this needs to be confirmed. Alternatively, they can record from non-infected CINs in the same slice preparations.

c. The time course of changes in firing rate following CNO addition is confusing. Why is the effect so delayed with the Gs DREADD? Please discuss.

d. Why is the effect of CNO sustained following CNO wash out in B1 and B2? Please address in results or discussion.

e. Are there changes in baseline CIN firing without CNO in these animals? (in other words, are the DREADDs leaky)? From the recordings shown it appears that the baseline (pre-CNO) firing is different in cells from different animals.

---- Basic parameters associated with excitability such as rheobase, firing-current relationships, IV curves, input resistance etc. are not included. Without such measurements, it is difficult to assess the effect of the manipulations on MSN excitability per se. Such controls should be included.

2. Several aspects of the methods need to be more explicitly delineated.

It is particularly important that the authors do a better job of conveying the methods employed. The paradigms used to assess excitability of MSNs are not explained anywhere in the manuscript. This is fundamentally important, as MSNs in the slice are normally quiet, and the reader needs to know precisely how the activity is driven.

The authors say they use a ramp activation protocol but more detail is required for the reproducibility of the reported findings and for such studies to be confirmed and/ or extended. As such in Figure 7, the "ramp" used in Fig 7B should be explicitly shown, and current or voltage levels at baseline should be included.

Absolute resting membrane potential in Fig 7C should be shown, preferably before and after CNO wash in (or other drugs).

3. Data presentation clarified

The full extent of the population data should be shown for all electrophysiological recordings, including either all of the data for individual cells. Ideally, the bar graphs should be replaced with scatter plots +/- box plots, so that the median, distribution, range etc could be directly accessible to the reader. At the very least the authors must provide the mean plus delineation of the full range of the data and precise description of how the error was calculated.

Minor critiques:

1. The word transduction should be used to describe cells that have been infected with a virus as opposed to transfected.

2. There are multiple grammar problems, please have someone edit the manuscript carefully.
